# Associations of Nutrient Patterns with the Prevalence of Metabolic Syndrome: Results from the Baseline Data of the Japan Multi-Institutional Collaborative Cohort Study

**DOI:** 10.3390/nu11050990

**Published:** 2019-04-30

**Authors:** Yuki Iwasaki, Kokichi Arisawa, Sakurako Katsuura-Kamano, Hirokazu Uemura, Mineko Tsukamoto, Yuka Kadomatsu, Rieko Okada, Asahi Hishida, Keitaro Tanaka, Megumi Hara, Toshiro Takezaki, Keiichi Shimatani, Etsuko Ozaki, Teruhide Koyama, Sadao Suzuki, Hiroko Nakagawa-Senda, Kiyonori Kuriki, Naoko Miyagawa, Aya Kadota, Hiroaki Ikezaki, Norihiro Furusyo, Isao Oze, Hidemi Ito, Haruo Mikami, Yohko Nakamura, Kenji Wakai

**Affiliations:** 1 Department of Preventive Medicine, Institute of Biomedical Sciences, Tokushima University Graduate School of Biomedical Sciences, Tokushima 770-8503, Japan; c201750006@tokushima-u.ac.jp (Y.I.); skamano@tokushima-u.ac.jp (S.K.-K.); uemura.hirokazu@tokushima-u.ac.jp (H.U.); 2 Department of Preventive Medicine, Nagoya University Graduate School of Medicine, Nagoya 466-8550, Japan; tsukamoto.mineko@b.mbox.nagoya-u.ac.jp (M.T.); yuka.kadomatsu@gmail.com (Y.K.); rieokada@med.nagoya-u.ac.jp (R.O.); a-hishi@med.nagoya-u.ac.jp (A.H.); wakai@med.nagoya-u.ac.jp (K.W.); 3 Department of Preventive Medicine, Faculty of Medicine, Saga University, Saga 849-8501, Japan; tanakake@cc.saga-u.ac.jp (K.T.); harameg@cc.saga-u.ac.jp (M.H.); 4 Department of International Island and Community Medicine, Kagoshima University Graduate School of Medical and Dental Sciences, Kagoshima 890-8544, Japan; takezaki@m.kufm.kagoshima-u.ac.jp; 5 Nurse Specific Medical Practice Training Center, Kagoshima University Hospital, Kagoshima 890-8520, Japan; shimanowski@gmail.com; 6 Department of Epidemiology for Community Health and Medicine, Kyoto Prefectural University of Medicine, Kyoto 602-8566, Japan; ozaki@koto.kpu-m.ac.jp (E.O.); tkoyama@koto.kpu-m.ac.jp (T.K.); 7 Department of Public Health, Nagoya City University Graduate School of Medical Sciences, Nagoya 467-8601, Japan; ssuzuki@med.nagoya-cu.ac.jp (S.S.); nakagawa@med.nagoya-cu.ac.jp (H.N.-S.); 8 Laboratory of Public Health, Division of Nutritional Sciences, School of Food and Nutritional Sciences, University of Shizuoka, Shizuoka 422-8526, Japan; kuriki@u-shizuoka-ken.ac.jp; 9 International Center for Nutrition and Information, National Institute of Health and Nutrition, National Institutes of Biomedical Innovation, Health and Nutrition, Tokyo 162-8636, Japan; naocom@belle.shiga-med.ac.jp; 10 Center for Epidemiologic Research in Asia, Shiga University of Medical Science, Otsu, Shiga 520-2192, Japan; ayakd@belle.shiga-med.ac.jp; 11 Department of Environmental Medicine and Infectious Disease, Graduate School of Medical Sciences, Kyushu University, Fukuoka 812-8582, Japan; ikezaki@gim.med.kyushu-u.ac.jp (H.I.); furusyo@gim.med.kyushu-u.ac.jp (N.F.); 12 Division of Cancer Epidemiology and Prevention, Aichi Cancer Center Research Institute, Nagoya 464-8681, Japan; i_oze@aichi-cc.jp; 13 Division of Cancer Information and Control, Aichi Cancer Center Research Institute, Nagoya 464-8681, Japan; hidemi@aichi-cc.jp; 14 Cancer Prevention Center, Chiba Cancer Center Research Institute, Chiba 260-8717, Japan; hmikami@chiba-cc.jp (H.M.); ynakamur@chiba-cc.jp (Y.N.)

**Keywords:** nutrient pattern, metabolic syndrome, factor analysis, cross-sectional study

## Abstract

The association between nutrient patterns and metabolic syndrome (MetS) has not been examined in a Japanese population. A cross-sectional study was performed on 30,108 participants (aged 35–69 years) in the baseline survey of the Japan Multi-Institutional Collaborative Cohort Study. Dietary intake was assessed using a 46-item food frequency questionnaire. MetS was diagnosed according to the Joint Interim Statement Criteria of 2009, using body mass index instead of waist circumference. Factor analysis was applied to energy-adjusted intake of 21 nutrients, and three nutrient patterns were extracted: Factor 1 (fiber, potassium and vitamins pattern); Factor 2 (fats and fat-soluble vitamins pattern); and Factor 3 (saturated fatty acids, calcium and vitamin B_2_ pattern). In multiple logistic regression analysis adjusted for sex, age, and other potential confounders, Factor 1 scores were associated with a significantly reduced odds ratio (OR) of MetS and all five components. Factor 2 scores were associated with significantly increased prevalence of MetS, obesity, and high blood pressure. Factor 3 scores were significantly associated with lower OR of MetS, high blood pressure, high serum triglycerides and low HDL cholesterol levels. Analysis of nutrient patterns may be useful to assess the overall quality of diet and its association with MetS.

## 1. Introduction

Metabolic syndrome (MetS) is a condition characterized by clustering of abdominal obesity, insulin resistance, high blood pressure, dyslipidemia, and high blood glucose levels, often accompanied by a proinflammatory and prothrombotic state [1,2]. MetS has become a public health concern not only in developed, but also developing countries because of its high frequency of occurrence [3]. According to the National Nutritional and Health Survey of 2016, the prevalence of MetS in Japan was estimated at 27.0% for men and 10.0% for women aged 20 years or older [4]. Prospective studies have shown that persons who have MetS are at increased risk of developing type 2 diabetes [5,6] and atherosclerotic cardiovascular diseases [5,7]. Therefore, primary prevention of MetS is important to prevent more severe health consequences. Although excessive energy intake and physical inactivity are major risk factors, diet quality may also contribute to MetS development [1].

There are several approaches to investigate the associations of diet with chronic diseases in epidemiologic studies. Dietary pattern analysis has been frequently used because foods and nutrients are consumed as an aggregate, and amounts of foods and nutrients are often highly correlated with each other, making it difficult to investigate the contribution of an individual food or nutrient separately [8,9]. A priori approach evaluates the degree of adherence to specific dietary patterns such as the Alternate Healthy Eating Index [10], Dietary Approach to Stop Hypertension (DASH) diet [11], and Mediterranean diet [12]. The latter two diets [13,14] were inversely associated with MetS. On the other hand, a posteriori approach extracts dietary patterns from data at hand. The most frequently used are principal component or factor analysis. A recent meta-analysis showed that a prudent/healthy pattern was inversely associated, while a Western/unhealthy pattern was positively associated, with MetS in cross-sectional studies, but not in cohort studies [15]. Nutrient pattern analysis extracts patterns from nutrient intake data instead of food intake. Recently, this approach has been applied to examine nutritional factors associated with MetS and fasting blood glucose levels in several studies [16,17,18,19].

In the present study, we investigated the associations of nutrient patterns with the prevalence of MetS in individuals who had participated in the baseline survey of the Japan Multi-Institutional Collaborative Cohort (J-MICC) Study.

## 2. Materials and Methods

### 2.1. Study Subjects

The population for the present study consisted of Japanese men and women aged 35 to 69 years who had participated in the baseline survey of the J-MICC Study. Details of the J-MICC Study have been described in a previous report [20]. Briefly, the aim of the J-MICC Study was to prospectively examine the interactions of lifestyle and genetic factors with the risk of chronic diseases. Written informed consent was obtained from each participant after a thorough explanation of the outline and purposes of this study. The study protocol was approved by the institutional review boards of Nagoya University School of Medicine (an affiliate of the present and former principal investigators, Drs. Kenji Wakai and Nobuyuki Hamajima) (IRB No. 939-13), Aichi Cancer Center Research Institute (affiliated with the former principal investigator, Dr. Hideo Tanaka) (IRB No. 2016-2-10), Tokushima University Hospital (IRB No. 466-2), and all other study centers (Faculty of Medicine, Saga University; Kagoshima University Graduate School of Medical and Dental Sciences; Kyoto Prefectural University of Medicine; Nagoya City University Graduate School of Medical Sciences; School of Food and Nutritional Sciences, University of Shizuoka; Shiga University of Medical Science; Graduate School of Medical Sciences, Kyushu University; and Chiba Cancer Center Research Institute).

**Figure 1:**
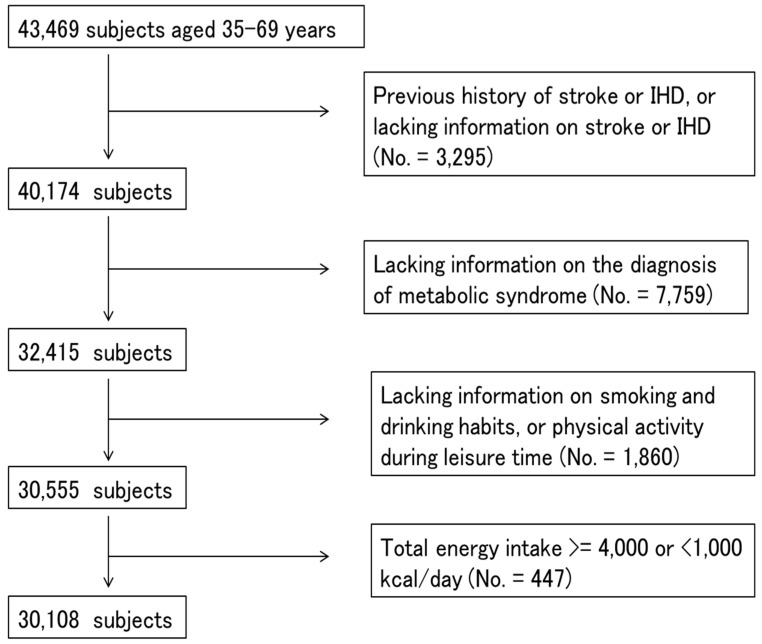
A flowchart showing the process for selecting the study subjects.

Participants from seven study centers that used the same questionnaires and performed blood examinations were included (No. = 43,469). We excluded participants who had a previous history of ischemic heart disease, stroke or lacked information on previous diseases (No. = 3295), who had missing values for items for the diagnosis of MetS (No. = 7759), or smoking and drinking habits, or physical activity (No. = 1860), or whose total energy intake was extremely high (≥4000 kcal/day) or low (<1000 kcal/day) (No. = 447) (Figure 1). Finally, a total of 30,108 participants (14,944 men and 15,164 women) were used for statistical analysis.

### 2.2. Questionnaire (Food Frequency Questionnaire (FFQ) and Covariates)

Study subjects were asked to fill out a self-administered questionnaire about dietary habits, current and previous diseases, medication and supplement consumption, physical activity, ways of coping with stress, and smoking and drinking habits. A validated short FFQ was used for dietary evaluation [21,22]. Participants were asked how often they had consumed 46 foods and beverages over the past year. Consumption of rice, bread, and noodles at breakfast, lunch, and dinner were divided into six categories from rarely to every day. For the other 43 foods and beverages, the intake frequency was categorized into eight categories from rarely to ≥3 times per day. Information on the portion size was collected for staple foods. Intake of total energy and 26 nutrients (protein, fat, carbohydrate, soluble dietary fiber, insoluble dietary fiber, total dietary fiber, saturated fatty acids, monounsaturated fatty acid, n-3 polyunsaturated fatty acids, n-3 highly unsaturated fatty acids, n-6 polyunsaturated fatty acids, polyunsaturated fatty acids, cholesterol, potassium, iron, calcium, retinol, carotene, vitamin D, E, B_1_, B_2_, C, folate, sodium, energy from alcohol) were computed using a program developed by the Department of Public Health, Nagoya City University School of Medicine. A validation study was performed by comparing the intake of total energy and 26 nutrients estimated using the FFQ and 3 day-weighed diet records. Deattenuated, log-transformed, and energy-adjusted correlation coefficients for intake of total energy and 26 nutrients ranged from 0.10 to 0.86 [21]. The correlation coefficients were high for carbohydrate (0.86), vitamin D (0.65), saturated fatty acids (0.64), fat (0.62) and iron (0.58), and low for n-6 polyunsaturated fatty acids (0.12), cholesterol (0.13), soluble dietary fiber (0.25), vitamin B_1_ (0.26) and vitamin A (0.27) [21]. This FFQ also showed high one-year interval reproducibility for consumption of foods and nutrients [22].

Physical activity during leisure time was evaluated using a self-administered questionnaire similar to a short format of the International Physical Activity Questionnaire [23]. Total physical activity levels were estimated by multiplying the frequency (five categories from never to ≥5 times/week) and average duration (six categories from ≤30 min to ≥4 h) of light (such as walking and golf at 3.4 metabolic equivalents (METs)), moderate (such as jogging, swimming, and dancing at 7.0 METs), and vigorous intensity exercises (such as marathon running at 10.0 METs). The three levels of exercises were summed and expressed as MET-hours/week. Height and weight were also measured, and body mass index (BMI) was calculated as weight (kg) divided by the square of height (m^2^). Smoking status was classified as current smoker, past smoker, and never, and drinking status was categorized as current drinker, past drinker, and never. School career was classified as elementary school/junior high school, high school, professional school, junior college/technical college, university/college, graduate school, and others.

### 2.3. Diagnosis of Metabolic Syndrome

MetS was diagnosed according to the Joint Interim Statement Criteria of 2009 [2]. Since waist circumference was not measured for every study subject, BMI was used. BMI is closely correlated with abdominal circumference, and the most accurate BMI cut-off point for large abdominal circumference (90 cm for men and 80 cm for women, the cut-off point for the diagnosis of MetS for Asians [2]) was approximately 25 kg/m^2^ [24]. Subjects with three or more of the following five conditions were defined as having MetS: BMI ≥25 kg/m^2^; blood pressure ≥130/85 mmHg or treatment for hypertension, serum HDL cholesterol level <40 mg/dL for men and <50 mg/dL for women; serum triglycerides ≥150 mg/dL; and fasting blood glucose level ≥100 mg/dL or treatment for diabetes.

### 2.4. Statistical Analysis

Data on nutrient intake were natural log-transformed and energy-adjusted using the residual method. Factor analysis (principal component analysis) with varimax rotation was applied to 21 nutrients after excluding five redundant nutrients. The number of nutrient patterns extracted was determined by eigenvalues, a scree plot and their interpretability.

Continuous variables are shown as the means ± standard deviations or medians (25th and 75th percentiles), and categorical variables are expressed as the counts and proportions. We examined the differences in participants’ characteristics according to the presence or absence of MetS using the *t*-test, Wilcoxon’s rank sum test, or chi-square test.

Logistic regression analysis was performed to evaluate the associations of the quartiles of nutrient pattern scores with the prevalence of MetS and its components, after adjustment for potential confounding variables. The first quartile was used as a reference to estimate odds ratios (OR) and their profile likelihood 95% confidence intervals (CI). Model 1 adjusted for age, sex and study center, and model 2 adjusted for variables included in model 1 and total energy intake (quartile), physical activity (quartile), drinking (three categories) and smoking (three categories) habits, and school career (eight categories). Model 3 adjusted for covariates included in model 2 and BMI (quartile). Finally, stratified analyses according to BMI and sex were also performed. Trends across the quartiles were examined by using ordinal categorical variables and a likelihood ratio test. All data analyses were performed using FACTOR, LOGISTIC and other procedures of SAS version 8.2 (SAS Institute, Inc. Cary, NC, USA).

## 3. Results

Table 1 shows the characteristics of the study subjects according to the presence of MetS. The prevalence of MetS was 22.6% for men and 10.6% for women. Subjects who had MetS were older, more likely to be men, current or past-smokers, current drinkers, and consumed more total energy. There was no significant difference in the physical activity level in leisure time among subjects who had MetS and those who did not. Table 1 also shows the median intake of total fat and 21 nutrients used for factor analysis according to the presence of MetS.

In factor analysis, three nutrient patterns were extracted (Table 2). In this analysis, factors with eigenvalues >1.5 were retained because this cut-off yielded more interpretable nutrient patterns. The first factor (Factor 1) explained by far the largest proportion of total variance in nutritional intake (45%), followed by the second (Factor 2, 12%) and third (Factor 3, 9%). Factor 1 was named the “fiber, potassium and vitamins pattern” because of the high factor loadings for folate, insoluble dietary fiber, carotene, soluble dietary fiber, iron, vitamin C, and potassium. Factor 2 was labeled the “fats and fat-soluble vitamins pattern” because it was positively correlated with monounsaturated fatty acid, *n*-3 fatty acids, *n*-6 fatty acids, and vitamin E. Factor 3 was named the “saturated fatty acids, calcium and vitamin B_2_ pattern” because of a positive correlation with saturated fatty acids, calcium, vitamin B_2_, and protein, and a negative correlation with carbohydrate. When factor analysis was performed for sex separately, the extracted nutrient patterns were similar (Table S1). Therefore, we adopted combined analysis of men and women. Table 1 presents the mean factor scores among subjects who had MetS and those who did not.

Table 3, Table 4 and Table 5 show the associations of three nutrient pattern scores with the prevalence of MetS and its components. After adjustment for sex, age, study site, physical activity, total energy intake, smoking and drinking habits, and school career (Model 2), Factor 1 scores were inversely and significantly associated with the prevalence of MetS (Q1 versus Q4, OR = 0.69, 95% CI 0.63–0.77, *p* for trend <0.001) and all five components (Table 3). On the other hand, Factor 2 scores were significantly associated with increased OR of MetS (Q1 versus Q4, OR = 1.27, 95% CI 1.17–1.39, *p* for trend <0.001), obesity, and high blood pressure in Model 2 (Table 4). To examine whether the associations of Factor 2 scores with four components of MetS (except for obesity) were mediated by obesity, BMI was further adjusted in Model 3. The OR for high blood pressure became somewhat lower, but remained statistically higher than 1.00. Factor 3 scores were associated with significantly reduced OR of MetS (Q1 versus Q4, OR = 0.87, 95% CI 0.79–0.95, *p* for trend <0.001), high blood pressure, high serum triglycerides, and low serum HDL cholesterol levels (Table 5). After adjusting for BMI, there were essentially no changes in the OR of each component.

When logistic regression analysis was performed using nutrient pattern scores as continuous variables, the *p*-values did not differ greatly from the *p* for trend shown in Table 3, Table 4 and Table 5 (Tables S2–S4). In a subgroup with BMI < 25 kg/m^2^ (total No. = 22,748, No. of MetS = 1194), only Factor 1 scores were significantly associated with reduced OR of MetS. In a subgroup with BMI ≥25 kg/m^2^ (total No. = 7360, No. of MetS = 3782), Factor 1 and 3 scores were inversely associated, while Factor 2 scores were positively associated with the prevalence of MetS (Table 6). When stratified by sex, the associations of Factor 1, 2, and 3 scores and MetS were similar for men and women.

## 4. Discussion

In the present study, Factor 1 scores, which were positively correlated with intake of folate, insoluble and soluble dietary fiber, carotene, iron, vitamin C and potassium, showed significant inverse associations with MetS and all its components. These seven nutrients are abundantly contained in vegetables and fruits. Therefore, the present results are consistent with observations that the prudent/healthy diet pattern, characterized by high intake of vegetables, fruits, legumes and cereals, was inversely associated with MetS in cross-sectional [15,25,26,27,28] and cohort studies [29,30]. A vitamins and trace elements pattern, extracted from 24-h dietary recall, was also reported to be inversely associated with the prevalence of MetS in the National Health and Nutrition Examination Survey [19]. Among the seven nutrients, insoluble dietary fiber and potassium were the most strongly associated with reduced OR of MetS (Table S5). Insoluble dietary fiber is known to prolong the intestinal transit time and increase post-meal satiety [31], as well as reducing insulin resistance [32]. After adjustment for BMI, insoluble dietary fiber remained inversely associated with low serum HDL cholesterol levels (Table S5), but we could not find plausible biological mechanisms. A recent meta-analysis showed that high dietary potassium intake was associated with lower prevalence of MetS [33]. A cross-sectional study conducted in China also reported an inverse association between serum potassium concentration and the prevalence of MetS [34]. Regarding this MetS component, it is established that increased potassium intake reduces blood pressure by counteracting the effects of sodium [35]. The other nutrients positively correlated with Factor 1 may have also contributed to the inverse association with MetS, through mechanisms such as the gel-forming effects of soluble dietary fiber to reduce postprandial blood glucose [31], and the anti-oxidative effects of vitamin C.

Factor 2 scores, which were positively correlated with monounsaturated fatty acid, n-3 and n-6 polyunsaturated fatty acids, and vitamin E, were positively associated with the prevalence of MetS, obesity, and high blood pressure. We think that Factor 2 reflects the overall consumption of a high fat diet because this pattern was positively correlated with deep fried foods, stir fried foods, mayonnaise, beef/pork, ham/sausage/salami/bacon, and fish. A high fat diet has high energy density, so it may lead to excessive energy intake over the long term. When BMI was additionally adjusted, the association of Factor 2 scores with high blood pressure was attenuated, but was still significantly increased for Q3 and Q4. This suggested that the association between Factor 2 scores and high blood pressure was partially mediated by obesity. In diet pattern analysis, the Western/unhealthy pattern, characterized by high intake of refined grains, red meat, processed meat, and fried foods, has been reported to be positively associated with MetS [15,25,36]. Factor 2 extracted in our study resembled the Western/unhealthy diet pattern, but was somewhat different in terms of the positive correlation with n-3 polyunsaturated fatty acids and fish intake, and a low correlation with saturated fatty acids. The association between Factor 2 and refined grains was unknown, since the distinction between whole/refined grains was not taken into account in our FFQ.

Factor 3 scores were significantly associated with lower OR of MetS and its three components, except for obesity and high blood glucose. Among three nutrients closely correlated with Factor 3 (saturated fatty acid, calcium, and vitamin B_2_), calcium was most strongly associated with a reduced OR of MetS (Table S6). After adjustment for Ca, saturated fatty acid intake and vitamin B_2_ were not independently associated with MetS. With regard to food items, milk, yogurt, beef and pork, and eggs were positively correlated with Factor 3 scores (data not shown), which was considered a reason for the high factor loading of saturated fatty acid on Factor 3. Cross-sectional studies showed inverse associations between calcium intake and MetS in U.S. women [37] and Korean women with low calcium intake (mean = 331 mg/day) [38]. Calcium density was also significantly associated with a lower MetS incidence rate [39]. Consumption of dairy products, a major source of calcium, has been reported to be inversely associated with the MetS incidence rate [36,39], despite some inconsistencies [40]. Mechanisms for the beneficial effects of calcium on lipid profiles include formation of soaps with fatty acids and increased fecal fat excretion, as well as its preventive effects on high blood pressure including reduced intracellular calcium concentration and vascular resistance, especially in individuals with low calcium intake [41]. In the present study, extracted nutrient patterns were different from those reported for Chinese [16], Iranian [17], and African populations [18]. However, two of the three extracted nutrient patterns were closely related to well-known dietary patterns (healthy/prudent and high fat/Western), which were also extracted in our previous study using the same FFQ [42]. One advantage of using nutrient patterns may be that interpretation of underlying biological mechanisms is more straightforward than dietary patterns.

In a stratified analysis according to BMI, Factor 1 scores were associated with lower OR of MetS among subjects with normal weight. On the other hand, Factor 1 and 3 scores were inversely associated, and Factor 2 scores were positively associated with the prevalence of MetS among overweight/obese subjects. This kind of analysis may be helpful to clarify the modifiable lifestyle factors associated with transition from metabolically healthy to unhealthy status according to boy weight [43]. The present results were in line with those recently reported for healthy dietary pattern characterized by high intake of vegetables, fruits and fish [44] and Mediterranean diet [45] associated with metabolically healthy obese status.

The strengths of the present study include the large number of study subjects, and use of a validated FFQ. On the other hand, there are also several limitations. First, the number of subjects excluded from the study was large (No. = 13,361). We compared the characteristics of those who were excluded and not excluded from the final analysis. The most striking difference was the availability of data for the diagnosis of MetS, especially high fasting blood glucose. It is unknown how the results were affected by the exclusion of a relatively large number of study subjects. Second, in cross-sectional studies, the temporal relationship between nutrient intake and onset of MetS is unclear. Third, since nutrient intake was assessed using an FFQ, some degree of measurement error is inevitable. However, only a small number of nutrients showed low validity in our FFQ [21], and nutrient patterns 1-3 were not heavily loaded by these nutrients. Fourth, because of a lack of data on waist circumference, BMI was alternatively used for the diagnosis of MetS. Therefore, some subjects with high muscle mass or excess subcutaneous fat may have mistakenly been judged as having abdominal obesity, while subjects with normal BMI but high abdominal circumference may have been overlooked. When the BMI cut-off point of 25 kg/m^2^ was used, the sensitivity and specificity of diagnosing high abdominal circumference were 83% and 82% for men (No. = 633), and 45% and 98% for women (No. = 623), respectively, in the sub-sample of the J-MICC Study in Tokushima Prefecture. Fifth, nutrient patterns may be specific to the study population, and interpretation may be subjective, as is the case for the posteriori dietary pattern analysis. This may make it difficult to generalize the results to other ethnic populations.

In conclusion, the fiber, potassium and vitamins pattern, and saturated fatty acids, calcium and vitamin B_2_ pattern were associated with a reduced OR of MetS, while a fats and fat-soluble vitamins pattern was associated with an increased OR of MetS. Analysis of nutrient patterns may be useful to assess the overall diet and its association with MetS, and to set dietary guidelines for MetS prevention.

## Figures and Tables

**Table 1:** Background characteristics of the study subjects according to the presence of metabolic syndrome.

	Metabolic Syndrome		
	+ (No. = 4976)	− (No. = 25132)	*p*-Value
Age (years) ^a^	56.7 ± 8.7	53.9 ± 9.7	<0.001
Sex ^b^			
Men	3371 (67.7)	11,573 (46)	<0.001
⬜Women	1605 (32.3)	13,559 (54)	
Study site ^b^			
⬜Okazaki	951 (19.1)	5213 (20.7)	<0.001
⬜Shizuoka, Daiko	711 (14.3)	3968 (15.8)	
⬜Takashima	257 (5.2)	1698 (6.8)	
Kyoto	833 (16.7)	4764 (19.0)	
Kagoshima	1407 (28.3)	4896 (19.5)	
⬜Tokushima	196 (3.9)	982 (3.9)	
⬜Sakuragaoka	621 (12.5)	3611 (14.4)	
Smoking habit ^b^			
⬜Current	992 (19.9)	3958 (15.8)	<0.001
Past	1618 (32.5)	5636 (22.4)	
⬜No	2366 (47.6)	15,538 (61.8)	
Drinking habit ^b^			
⬜Current	3147 (63.2)	14,221 (56.6)	<0.001
Past	84 (1.7)	420 (1.7)	
⬜ No	1745 (35.1)	10,491 (41.7)	
Exercise during leisure time (MET-hours/week) ^c^	6.45 (0.425, 17.85)	5.675 (0.425, 17.925)	0.657
Body mass index (kg/m^2^) ^a^	26.6 ± 3.2	22.3 ± 2.8	<0.001
Systolic blood pressure (mmHg) ^c^	136 (129, 146)	122 (110, 133)	<0.001
Diastolic blood pressure (mmHg) ^c^	84 (78, 90)	75 (68, 82)	<0.001
Serum triglycerides (mg/dL) ^c^	172 (121, 228)	85 (63, 117)	<0.001
Serum HDL cholesterol (mg/dL) ^c^	49 (43, 60)	65 (55, 77)	<0.001
Fasting blood glucose (mg/dL) ^c^	105 (98, 116)	92 (87, 98)	<0.001
Energy intake(kcal/day) ^a^	1800 ± 370	1727 ± 345	<0.001
Protein (g/day) ^c^	53 (46, 60)	52 (46, 59)	0.015
Fat (g/day) ^c^	42 (35, 49)	43 (37, 50)	<0.001
Carbohydrate (g/day) ^c^	248 (212, 291)	237 (206, 278)	<0.001
Soluble dietary fiber (g/day) ^c^	1.7 (1.4, 2.1)	1.8 (1.5, 2.2)	<0.001
Insoluble dietary fiber (g/day) ^c^	6.9 (5.8, 8.3)	7.4 (6.2, 8.8)	<0.001
Saturated fatty acids (g/day) ^c^	10.5 (9.0, 12.2)	11.0 (9.3, 12.8)	<0.001
Monounsaturated fatty acids (g/day) ^c^	15.5 (13.3, 18.4)	15.7 (13.5, 18.4)	<0.001
*n*-3 polyunsaturated fatty acids (mg/day) ^c^	2164 (1800, 2595)	2164 (1847, 2539)	0.859
*n*-6 polyunsaturated fatty acids (mg/day) ^c^	10,528 (8811, 12,806)	10,679 (9032, 12,758)	<0.001
Cholesterol (mg/day) ^c^	229 (184, 289)	233 (185, 288)	0.248
Potassium (mg/day) ^c^	1980 (1715, 2268)	2044 (1785, 2356)	<0.001
Calcium (mg/day) ^c^	464 (382, 568)	498 (404, 599)	<0.001
Iron (mg/day) ^c^	6.8 (5.6, 8.2)	6.8 (5.7, 8.2)	0.002
Retinol EQ (μg/day) ^c^	859 (574, 1134)	872 (615, 1152)	<0.001
Carotene (μg/day) ^c^	2567 (1941, 3438)	2835 (2114, 3760)	<0.001
Vitamin D (μg/day) ^c^	6.9 (4.7, 8.6)	6.9 (4.7, 8.3)	0.005
Vitamin E (mg/day) ^c^	7.6 (6.5, 8.9)	7.8 (6.8, 9.2)	<0.001
Vitamin B_1_ (mg/day) ^c^	0.63 (0.58, 0.69)	0.64 (0.59, 0.69)	0.021
Vitamin B_2_ (mg/day) ^c^	1.03 (0.87, 1.20)	1.06 (0.90, 1.24)	<0.001
Vitamin C (mg/day) ^c^	87 (67, 107)	91 (71, 114)	<0.001
Folate (μg/day) ^c^	307 (250, 373)	318 (261, 387)	<0.001
Sodium (mg/day) ^c^	1667 (1414, 2015)	1683 (1433, 1976)	0.598
Nutritional pattern score ^a^			
⬜Factor 1	−0.16 ± 1.00	0.03 ± 1.00	<0.001
⬜Factor 2	−0.01 ± 1.02	0.00 ± 1.00	0.637
⬜Factor 3	−0.10 ± 0.99	0.02 ± 1.00	<0.001

^a^ Mean + S.D, *t*-test; ^b^ Number of subjects (%), chi-square test; ^c^ Median (25%, 75%), Wilcoxon’s rank sum test.

**Table 2:** Factor loading matrix for selected nutrient patterns.

	Factor 1	Factor 2	Factor 3
Protein	0.36	0.43	0.64
Carbohydrate	0.09	−0.28	−0.59
Soluble dietary fiber	0.82	0.19	0.19
Insoluble dietary fiber	0.84	0.22	0.17
Saturated fatty acids	0.13	0.17	0.86
Monounsaturated fatty acids	0.12	0.92	0.17
*n*-3 polyunsaturated fatty acids	0.24	0.86	0.16
*n*-6 polyunsaturated fatty acids	0.28	0.80	0.07
Cholesterol	0.15	0.40	0.39
Potassium	0.76	0.15	0.31
Calcium	0.45	−0.05	0.75
Iron	0.81	0.19	0.17
Retinol	0.65	0.19	0.11
Carotene	0.82	0.24	0.06
Vitamin D	0.40	0.35	0.27
Vitamin E	0.49	0.78	0.09
Vitamin B_1_	0.10	0.55	0.43
Vitamin B_2_	0.52	0.04	0.71
Vitamin C	0.80	0.13	0.08
Folate	0.88	0.17	0.07
Sodium	0.47	0.14	0.17
Cumulative variance explained	0.45	0.57	0.66

**Table 3:** Associations of Factor 1 (fiber, potassium and vitamins pattern) scores with metabolic syndrome and its components.

	Q1 (No. = 7527)	Q2 (No. = 7527)	Q3 (No. = 7527)	Q4 (No. = 7527)	
	OR	OR (95% CI)	OR (95% CI)	OR (95% CI)	*p* for Trend
Metabolic syndrome					
Molel 1 ^a^	1.00	0.78 (0.72–0.85)	0.72 (0.66–0.79)	0.66 (0.60–0.73)	<0.001
Model 2 ^b^	1.00	0.80 (0.73–0.87)	0.74 (0.68–0.81)	0.69 (0.63–0.77)	<0.001
Obesity					
Model 1	1.00	0.81 (0.76–0.88)	0.81 (0.75–0.87)	0.76 (0.70–0.83)	<0.001
Model 2	1.00	0.81 (0.75–0.87)	0.80 (0.74–0.86)	0.75 (0.69–0.82)	<0.001
High blood pressure					
Model 1	1.00	0.83 (0.77–0.89)	0.78 (0.73–0.84)	0.73 (0.68–0.79)	<0.001
Model 2	1.00	0.82 (0.76–0.88)	0.78 (0.72–0.84)	0.74 (0.68–0.80)	<0.001
Model 3 ^c^	1.00	0.85 (0.79–0.91)	0.81 (0.75–0.87)	0.78 (0.72–0.85)	<0.001
High serum triglycerides					
Model 1	1.00	0.84 (0.77–0.90)	0.78 (0.72–0.85)	0.67 (0.61–0.73)	<0.001
Model 2	1.00	0.87 (0.80–0.94)	0.83 (0.76–0.91)	0.73 (0.66–0.80)	<0.001
Model 3	1.00	0.90 (0.83–0.98)	0.87 (0.80–0.95)	0.77 (0.70–0.85)	<0.001
Low HDL cholesterol					
Model 1	1.00	0.84 (0.75–0.95)	0.88 (0.78–0.99)	0.77 (0.68–0.88)	<0.001
Model 2	1.00	0.87 (0.78–0.99)	0.92 (0.82–1.04)	0.81 (0.71–0.92)	0.006
Model 3	1.00	0.91 (0.81–1.03)	0.97 (0.85–1.09)	0.87 (0.76–0.99)	0.09
High blood glucose					
Model 1	1.00	0.88 (0.82–0.95)	0.80 (0.74–0.86)	0.76 (0.70–0.82)	<0.001
Model 2	1.00	0.89 (0.83–0.96)	0.82 (0.75–0.88)	0.78 (0.72–0.85)	<0.001
Model 3	1.00	0.92 (0.85–0.99)	0.84 (0.78–0.91)	0.82 (0.76–0.90)	<0.001

OR, odds ratio; CI, confidence interval; ^a^ Adjusted for sex, age, and study site; ^b^ Additionally adjusted for smoking and drinking habits, physical activity level, total energy intake, and school career; ^c^ Additionally adjusted for body mass index.

**Table 4:** Associations of Factor 2 (fats and fat-soluble vitamins pattern) scores with metabolic syndrome and its components.

	Q1 (No. = 7527)	Q2 (No. = 7527)	Q3 (No. = 7527)	Q4 (No. = 7527)	
	OR	OR (95% CI)	OR (95% CI)	OR (95% CI)	*p* for Trend
Metabolic syndrome					
Molel 1 ^a^	1.00	1.11 (1.02–1.21)	1.06 (0.97–1.16)	1.27 (1.16–1.38)	<0.001
Model 2 ^b^	1.00	1.11 (1.02–1.21)	1.06 (0.97–1.16)	1.27 (1.17–1.39)	<0.001
Obesity					
Molel 1	1.00	1.00 (0.93–1.08)	1.06 (0.99–1.15)	1.26 (1.16–1.36)	<0.001
Model 2	1.00	1.01 (0.94–1.09)	1.08 (1.00–1.16)	1.27 (1.18–1.38)	<0.001
High blood pressure					
Molel 1	1.00	1.07 (1.00–1.14)	1.13 (1.06–1.21)	1.21 (1.13–1.30)	<0.001
Model 2	1.00	1.07 (1.00–1.14)	1.13 (1.05–1.21)	1.22 (1.13–1.31)	<0.001
Model 3 ^c^	1.00	1.06 (0.99–1.14)	1.11 (1.03–1.19)	1.15 (1.07–1.24)	<0.001
High serum triglycerides					
Molel 1	1.00	1.08 (0.99–1.17)	1.06 (0.98–1.15)	1.10 (1.01–1.19)	0.05
Model 2	1.00	1.06 (0.98–1.15)	1.06 (0.97–1.15)	1.09 (1.00–1.18)	0.07
Model 3	1.00	1.05 (0.97–1.15)	1.04 (0.95–1.13)	1.01 (0.93–1.10)	0.89
Low HDL cholesterol					
Molel 1	1.00	1.07 (0.96–1.20)	1.01 (0.89–1.13)	0.98 (0.87–1.10)	0.48
Model 2	1.00	1.09 (0.97–1.22)	1.05 (0.93–1.18)	1.01 (0.90–1.14)	1.00
Model 3	1.00	1.08 (0.96–1.22)	1.03 (0.91–1.16)	0.95 (0.84–1.07)	0.27
High blood glucose					
Molel 1	1.00	1.02 (0.95–1.10)	0.98 (0.91–1.06)	1.10 (1.02–1.18)	0.05
Model 2	1.00	1.02 (0.95–1.10)	0.98 (0.91–1.05)	1.09 (1.02–1.18)	0.07
Model 3	1.00	1.02 (0.94–1.09)	0.96 (0.89–1.04)	1.04 (0.97–1.12)	0.60

OR, odds ratio; CI, confidence interval; ^a^ Adjusted for sex, age, and study site; ^b^ Additionally adjusted for smoking and drinking habits, physical activity level, total energy intake, and school career; ^c^ Additionally adjusted for body mass index.

**Table 5:** Associations of Factor 3 (saturated fatty acids, calcium and vitamin B2 pattern) scores with metabolic syndrome and its components.

	Q1 (No. = 7527)	Q2 (No. = 7527)	Q3 (No. = 7527)	Q4 (No. = 7527)	
	OR	OR (95% CI)	OR (95% CI)	OR (95% CI)	*p* for Trend
Metabolic syndrome					
Molel 1 ^a^	1.00	1.01 (0.93–1.10)	0.87 (0.80–0.95)	0.83 (0.76–0.91)	<0.001
Model 2 ^b^	1.00	1.03 (0.94–1.12)	0.90 (0.82–0.98)	0.87 (0.79–0.95)	<0.001
Obesity					
Model 1	1.00	1.03 (0.96–1.11)	0.96 (0.89–1.03)	0.92 (0.86–1.00)	0.01
Model 2	1.00	1.05 (0.97–1.13)	0.98 (0.91–1.06)	0.96 (0.88–1.03)	0.12
High blood pressure					
Model 1	1.00	0.95 (0.89–1.02)	0.89 (0.83–0.96)	0.84 (0.79–0.90)	<0.001
Model 2	1.00	0.95 (0.88–1.02)	0.89 (0.83–0.95)	0.85 (0.79–0.91)	<0.001
Model 3 ^c^	1.00	0.93 (0.87–1.00)	0.87 (0.81–0.94)	0.84 (0.78–0.90)	<0.001
High serum triglycerides					
Model 1	1.00	0.98 (0.90–1.06)	0.86 (0.79–0.93)	0.87 (0.80–0.95)	<0.001
Model 2	1.00	0.99 (0.91–1.07)	0.89 (0.81–0.96)	0.91 (0.83–0.99)	0.004
Model 3	1.00	0.97 (0.89–1.05)	0.87 (0.80–0.95)	0.90 (0.83–0.98)	0.002
Low HDL cholesterol					
Model 1	1.00	0.88 (0.79–0.98)	0.79 (0.71–0.89)	0.72 (0.64–0.81)	<0.001
Model 2	1.00	0.93 (0.83–1.04)	0.86 (0.77–0.97)	0.80 (0.71–0.90)	<0.001
Model 3	1.00	0.92 (0.82–1.03)	0.86 (0.76–0.96)	0.79 (0.70–0.90)	<0.001
High blood glucose					
Model 1	1.00	1.04 (0.96–1.11)	1.01 (0.94–1.09)	0.99 (0.92–1.06)	0.59
Model 2	1.00	1.04 (0.97–1.12)	1.02 (0.95–1.10)	1.01 (0.93–1.09)	1.00
Model 3	1.00	1.03 (0.96–1.11)	1.02 (0.94–1.10)	1.00 (0.93–1.08)	0.97

OR, odds ratio; CI, confidence interval; ^a^ Adjusted for sex, age, and study site; ^b^ Additionally adjusted for smoking and drinking habits, physical activity level, total energy intake, and school career; ^c^ Additionally adjusted for body mass index.

**Table 6:** Associations of nutrient pattern scores with metabolic syndrome according to body mass index.

	**Q1 (No. = 5687)**	**Q2 (No. = 5687)**	**Q3 (No. = 5687)**	**Q4 (No. = 5687)**	
	**OR**	**OR (95% CI)**	**OR (95% CI)**	**OR (95% CI)**	***p* for Trend**
BMI <25 kg/m^2^					
Factor 1 ^a^	1.00	0.86 (0.73–1.02)	0.71 (0.59–0.84)	0.70 (0.58–0.84)	<0.001
Factor 2 ^a^	1.00	1.07 (0.91–1.25)	0.95 (0.80–1.13)	1.05 (0.88–1.24)	0.95
Factor 3 ^a^	1.00	1.02 (0.87–1.20)	0.97 (0.82–1.15)	0.93 (0.78–1.10)	0.33
	**Q1 (No. = 1840)**	**Q2 (No. = 1840)**	**Q3 (No. = 1840)**	**Q4 (No. = 1840)**	
	**OR**	**OR (95% CI)**	**OR (95% CI)**	**OR (95% CI)**	***p* for Trend**
BMI ≥25 kg/m^2^					
Factor 1	1.00	0.92 (0.80–1.06)	0.85 (0.74–0.98)	0.81 (0.70–0.94)	0.003
Factor 2	1.00	1.20 (1.05–1.38)	1.09 (0.95–1.25)	1.19 (1.04–1.37)	0.05
Factor 3	1.00	1.00 (0.87–1.15)	0.87 (0.76–0.99)	0.81 (0.71–0.93)	<0.001

OR, odds ratio; CI, confidence interval; ^a^ Adjusted for smoking and drinking habits, physical activity level, total energy intake, and school career.

## Supplementary Materials

The following are available online at https://www.mdpi.com/2072-6643/11/5/990/s1, Table S1: Factor loading matrix for extracted nutrient patterns in men and women, Table S2: Associations of Factor 1 scores with metabolic syndrome and its components, Table S3: Associations of Factor 2 scores with metabolic syndrome and its components, Table S4: Associations of Factor 3 scores with metabolic syndrome and its components, Table S5: Associations of insoluble dietary fiber and potassium with metabolic syndrome and its components, Table S6: Associations of saturated fatty acids, calcium and vitamin B_2_ with metabolic syndrome and its components.

## References

[1] Grundy S.M., Cleeman J.I., Daniels S.R., Donato K.A., Eckel R.H., Franklin B.A., Gordon D.J., Krauss R.M., Savage P.J., Smith S.C. (2005). Diagnosis and management of the metabolic syndrome: An American Heart Association/National Heart, Lung, and Blood Institute Scientific Statement. Circulation.

[2] Alberti K.G., Eckel R.H., Grundy S.M., Zimmet P.Z., Cleeman J.I., Donato K.A., Fruchart J.C., James W.P., Loria C.M., Smith S.C. (2009). Harmonizing the metabolic syndrome: A joint interim statement of the International Diabetes Federation Task Force on Epidemiology and Prevention; National Heart, Lung, and Blood Institute; American Heart Association; World Heart Federation; International Atherosclerosis Society; and International Association for the Study of Obesity. Circulation.

[3] Saklayen M.G. (2018). The Global epidemic of the metabolic syndrome. Curr. Hypertens. Rep..

[4] Ministry of Health, Labor and Welfare of Japan: A summary of the National Nutritional and Health Survey of 2016. https://www.mhlw.go.jp/bunya/kenkou/eiyou/dl/h28-houkoku.pdf.

[5] Sattar N., Gaw A., Scherbakova O., Ford I., O’Reilly D.S., Haffner S.M., Isles C., Macfarlane P.W., Packard C.J., Cobbe S.M. (2003). Metabolic syndrome with and without C-reactive protein as a predictor of coronary heart disease and diabetes in the West of Scotland Coronary Prevention Study. Circulation.

[6] Mukai N., Doi Y., Ninomiya T., Hata J., Yonemoto K., Iwase M., Iida M., Kiyohara Y. (2009). Impact of metabolic syndrome compared with impaired fasting glucose on the development of type 2 diabetes in a general Japanese population: The Hisayama study. Diabetes Care.

[7] Doi Y., Ninomiya T., Hata J., Yonemoto K., Arima H., Kubo M., Tanizaki Y., Iwase M., Iida M., Kiyohara Y. (2009). Proposed criteria for metabolic syndrome in Japanese based on prospective evidence: The Hisayama study. Stroke.

[8] Hu F.B. (2002). Dietary pattern analysis: A new direction in nutritional epidemiology. Curr. Opin. Lipidol..

[9] Michels K.B., Schulze M.B. (2005). Can dietary patterns help us detect diet-disease associations?. Nutr. Res. Rev..

[10] McCullough M.L., Willett W.C. (2006). Evaluating adherence to recommended diets in adults: The Alternate Healthy Eating Index. Public Health Nutr..

[11] Rai S.K., Fung T.T., Lu N., Keller S.F., Curhan G.C., Choi H.K. (2017). The Dietary Approaches to Stop Hypertension (DASH) diet, Western diet, and risk of gout in men: Prospective cohort study. BMJ.

[12] Trichopoulou A., Costacou T., Bamia C., Trichopoulos D. (2003). Adherence to a Mediterranean diet and survival in a Greek population. N. Engl. J. Med..

[13] Asghari G., Yuzbashian E., Mirmiran P., Hooshmand F., Najafi R., Azizi F. (2016). Dietary Approaches to Stop Hypertension (DASH) dietary pattern is associated with reduced incidence of metabolic syndrome in children and adolescents. J. Pediatr..

[14] Godos J., Zappalà G., Bernardini S., Giambini I., Bes-Rastrollo M., Martinez-Gonzalez M. (2017). Adherence to the Mediterranean diet is inversely associated with metabolic syndrome occurrence: A meta-analysis of observational studies. Int. J. Food Sci. Nutr..

[15] Rodríguez-Monforte M., Sánchez E., Barrio F., Costa B., Flores-Mateo G. (2017). Metabolic syndrome and dietary patterns: A systematic review and meta-analysis of observational studies. Eur. J. Nutr..

[16] Bian S., Gao Y., Zhang M., Wang X., Liu W., Zhang D., Huang G. (2013). Dietary nutrient intake and metabolic syndrome risk in Chinese adults: A case-control study. Nutr. J..

[17] Khayyatzadeh S.S., Moohebati M., Mazidi M., Avan A., Tayefi M., Parizadeh S.M., Ebrahimi M., Heidari-Bakavoli A., Azarpazhooh M.R., Esmaily H. (2016). Nutrient patterns and their relationship to metabolic syndrome in Iranian adults. Eur. J. Clin. Investig..

[18] Chikowore T., Pisa P.T., van Zyl T., Feskens E.J., Wentzel-Viljoen E., Conradie K.R. (2017). Nutrient patterns associated with fasting glucose and glycated haemoglobin levels in a black south African population. Nutrients.

[19] Mazidi M., Pennathur S., Afshinnia F. (2017). Link of dietary patterns with metabolic syndrome: Analysis of the National Health and Nutrition Examination Survey. Nutr. Diabetes.

[20] Hamajima N. (2007). J-MICC Study Group. The Japan Multi-Institutional Collaborative Cohort Study (J-MICC Study) to detect gene-environment interactions for cancer. Asian Pac. J. Cancer Prev..

[21] Tokudome Y., Goto C., Imaeda N., Hasegawa T., Kato R., Hirose K., Tajima K., Tokudome S. (2005). Relative validity of a short food frequency questionnaire for assessing nutrient intake versus three-day weighed diet records in middle-aged Japanese. J. Epidemiol..

[22] Imaeda N., Goto C., Tokudome Y., Hirose K., Tajima K., Tokudome S. (2007). Reproducibility of a short food frequency questionnaire for Japanese general population. J. Epidemiol..

[23] Claig C.L., Marshall A.L., Sjöström M., Bauman A.E., Booth M.L., Ainsworth B.E., Pratt M., Ekelund U., Yngve A., Sallis J.F. (2003). International physical activity questionnaire: 12-country reliability and validity. Med. Sci. Sports Exerc..

[24] Lauria M.W., Moreira L.M., Machado-Coelho G.L., Neto R.M., Soares M.M., Ramos A.V. (2013). Ability of body mass index to predict abnormal waist circumference: Receiving operating characteristics analysis. Diabetol. Metab. Syndr..

[25] Esmaillzadeh A., Kimiagar M., Mehrabi Y., Azadbakht L., Hu F.B., Willett W.C. (2007). Dietary patterns, insulin resistance, and prevalence of the metabolic syndrome in women. Am. J. Clin. Nutr..

[26] Panagiotakos D., Sitara M., Pitsavos C., Stefanadis C. (2007). The association between food patterns and the metabolic syndrome using principal components analysis: The ATTICA Study. J. Am. Diet. Assoc..

[27] Deshmukh-Taskar P.R., O’Neil C.E., Nicklas T.A., Yang S.J., Liu Y., Gustat J., Berenson G.S. (2009). Dietary patterns associated with metabolic syndrome, sociodemographic and lifestyle factors in young adults: The Bogalusa Heart Study. Public Health Nutr..

[28] Agodi A., Maugeri A., Kunzova S., Sochor O., Bauerova H., Kiacova N., Barchitta M., Vinciguerra M. (2018). Association of Dietary Patterns with Metabolic Syndrome: Results from the Kardiovize Brno 2030 Study. Nutrients.

[29] Duffey K.J., Steffen L.M., Van Horn L., Jacobs D.R. Jr., Popkin B.M. (2012). Dietary patterns matter: Diet beverages and cardiometabolic risks in the longitudinal Coronary Artery Risk Development in Young Adults (CARDIA) Study. Am. J. Clin. Nutr..

[30] Hassannejad R., Kazemi I., Sadeghi M., Mohammadifard N., Roohafza H., Sarrafzadegan N., Talaei M., Mansourian M. (2018). Longitudinal association of metabolic syndrome and dietary patterns: A 13-year prospective population-based cohort study. Nutr. Metab. Cardiovasc. Dis..

[31] Weickert M.O., Pfeiffer A.F.H. (2018). Impact of dietary fiber consumption on insulin resistance and the prevention of type 2 diabetes. J. Nutr..

[32] Robertson M.D., Wright J.W., Loizon E., Debard C., Vidal H., Shojaee-Moradie F., Russell-Jones D., Umpleby A.M. (2012). Insulin-sensitizing effects on muscle and adipose tissue after dietary fiber intake in men and women with metabolic syndrome. J. Clin. Endocrinol. Metab..

[33] Cai X., Li X., Fan W., Yu W., Wang S., Li Z., Scott E.M., Li X. (2016). Potassium and Obesity/Metabolic Syndrome: A Systematic Review and Meta-Analysis of the Epidemiological Evidence. Nutrients.

[34] Sun K., Su T., Li M., Xu B., Xu M., Lu J., Liu J., Bi Y., Ning G. (2014). Serum potassium level is associated with metabolic syndrome: A population-based study. Clin. Nutr..

[35] Filippini T., Violi F., D’Amico R., Vinceti M. (2017). The effect of potassium supplementation on blood pressure in hypertensive subjects: A systematic review and meta-analysis. Int. J. Cardiol..

[36] Lutsey P.L., Steffen L.M., Stevens J. (2008). Dietary intake and the development of the metabolic syndrome: The Atherosclerosis Risk in Communities study. Circulation.

[37] Liu S., Song Y., Ford E.S., Manson J.E., Buring J.E., Ridker P.M. (2005). Dietary calcium, vitamin D, and the prevalence of metabolic syndrome in middle-aged and older U.S. women. Diabetes Care.

[38] Shin S.K., Kim M.K., Lee Y.H., Shin D.H., Shin M.H., Chun B.Y., Choi B.Y. (2015). The cross-sectional relationship between dietary calcium intake and metabolic syndrome among men and women aged 40 or older in rural areas of Korea. Nutr. Res. Pract..

[39] Fumeron F., Lamri A., Abi Khalil C., Jaziri R., Porchay-Baldérelli I., Lantieri O., Vol S., Balkau B., Marre M., Data from the Epidemiological Study on the Insulin Resistance Syndrome (DESIR) Study Group (2011). Dairy consumption and the incidence of hyperglycemia and the metabolic syndrome: Results from a French prospective study, Data from the Epidemiological Study on the Insulin Resistance Syndrome (DESIR). Diabetes Care.

[40] Snijder M.B., van Dam R.M., Stehouwer C.D., Hiddink G.J., Heine R.J., Dekker J.M. (2008). A prospective study of dairy consumption in relation to changes in metabolic risk factors: The Hoorn Study. Obesity.

[41] Rice B.H., Cifelli C.J., Pikosky M.A., Miller G.D. (2011). Dairy components and risk factors for cardiometabolic syndrome: Recent evidence and opportunities for future research. Adv. Nutr..

[42] Arisawa K., Uemura H., Yamaguchi M., Nakamoto M., Hiyoshi M., Sawachika F., Katsuura-Kamano S. (2014). Associations of dietary patterns with metabolic syndrome and insulin resistance: A cross-sectional study in a Japanese population. J. Med. Investig..

[43] Schulze M.B. (2019). Metabolic health in normal-weight and obese individuals. Diabetologia.

[44] Slagter S.N., Corpeleijn E., van der Klauw M.M., Sijtsma A., Swart-Busscher L.G., Perenboom C.W.M., de Vries J.H.M., Feskens E.J.M., Wolffenbuttel B.H.R., Kromhout D. (2018). Dietary patterns and physical activity in the metabolically (un)healthy obese: The Dutch Lifelines cohort study. Nutr. J..

[45] Arenaza L., Huybrechts I., Ortega F.B., Ruiz J.R., De Henauw S., Manios Y., Marcos A., Julián C., Widhalm K., Bueno G. (2018). Adherence to the Mediterranean diet in metabolically healthy and unhealthy overweight and obese European adolescents: The HELENA study. Eur. J. Nutr..

